# White matter disconnection is related to age-related phonological deficits

**DOI:** 10.1007/s11682-019-00086-8

**Published:** 2019-04-02

**Authors:** Sara B. W. Troutman, Michele T. Diaz

**Affiliations:** Department of Psychology, Pennsylvania State University, 365 Moore Building, University Park, PA 16802 USA

**Keywords:** Diffusion tensor imaging, Phonology, Aging, Picture-word interference, Language production

## Abstract

Older adults have more language production difficulties than younger adults but display largely comparable language comprehension abilities. The Transmission Deficit Hypothesis suggests that production difficulties stem from an age-related increase in phonological signal transmission failures, while the semantic system, being more redundant than the phonological system, allows comprehension to be relatively preserved despite signal failures. Though the neural instantiation of the Transmission Deficit Hypothesis remains an open question, white matter represents one important factor to investigate. Metrics indicative of white matter connectivity across the brain, namely, Radial Diffusivity (RD) and Fractional Anisotropy (FA) have also been linked to age-related cognitive differences including naming difficulties. Using a Picture-Word Interference (PWI) task with 18 younger and 19 older healthy adults, we found that, across ages, better picture naming in the presence of phonological distractors was associated with lower RD across dorsal (*r* = −.35, *p* = .03), ventral (*r* = −.34, *p* = .04), and fronto-striatal (*r* = −.33, *p* = .04) tracts, and higher FA along dorsal tracts (*r* = .43, *p* = .008). The pattern of lower RD and higher FA, which is thought to reflect better white matter structure, points to the dorsal stream tracts as critical for performance on the PWI task. Moreover, the effects of RD and FA on performance were attenuated by the effect of age, reflecting the shared variance between age and white matter as it relates to language production ability.

**Electronic supplementary material:**

The online version of this article (10.1007/s11682-019-00086-8) contains supplementary material, which is available to authorized users.

## Introduction

Despite relative stability in language comprehension abilities across the lifespan, older adults, typically considered to be individuals over 60 years old, have more language production difficulties compared to younger adults (Burke and Shafto 2008). Cross-sectional work has revealed that older adults experience more tip-of-the-tongue states (Burke et al. 1991), produce more filler-words and pauses in speech (Kemper et al. 1992), and make more naming errors (Feyereisen 1997). Not only do older adults report that language problems are frequent and frustrating (Ossher et al. 2013), such language difficulties may also elicit negative social interactions (Ryan et al. 1995). As such, explaining the underlying mechanisms of age-related language production deficits has critical downstream consequences for the overall well-being of individuals as they age. Theoretical models and behavioral evidence suggest that age-related language production difficulties arise from phonological deficits, such as a slow or incomplete retrieval of a word’s sound form (Burke et al. 1991). However, the neural mechanisms underpinning such deficits remain an open question.

One potential neural substrate of age-related language deficits may be age-related differences in structural connectivity (i.e., white matter). Recent studies utilizing Diffusion Tensor Imaging (DTI) have indicated that, with age, water diffusion in the brain changes (Bennett et al. 2010; Madden et al. 2009; Davis et al. 2009). Although a number of factors influence DTI measurements, (i.e., axon diameter, permeability, density, hydration, etc.; Jones et al. 2013), a growing number of sources suggests that DTI signal differences can be attributed to differences in white matter, broadly speaking (Jones et al. 2013; Song et al. 2002; Takahashi et al. 2002; Pierpaoli et al. 1996; Basser and Pierpaoli 1996; Basser 1995). The two most commonly used DTI measures include Radial Diffusivity (RD) and Fractional Anisotropy (FA). RD reflects the second and third eigenvalues of the tensor and is interpreted to indicate water diffusion perpendicular to the modeled fiber with higher values indicating more diffusion across the fiber, and, thus, lower fiber integrity (Song et al. 2002; Jones et al. 2013; Basser 1995). FA reflects all three eigenvalues and therein the overall microstructure and shape of water diffusion. Higher FA values indicate more elliptical or constrained flow (i.e., higher fiber coherence) and lower values indicate more isotropic or less constrained flow (i.e., poorer fiber coherence; Behrens et al. 2003; Jones et al. 2013). Thus, RD and FA are inherently linked and should be interpreted together (Bennett et al. 2010; Jones et al. 2013). For instance, commonly observed, age-related differences include both higher RD (Davis et al. 2009; Madden et al. 2009) and lower FA (Davis et al. 2009; Gazes et al. 2016; Madden et al. 2009). Moreover, several studies have demonstrated that DTI indicators of white matter can influence brain function–behavior relationships in a host of cognitive tasks including processing speed, executive function, reasoning, and episodic memory (Fjell et al. 2017; Gazes et al. 2016; Hedden et al. 2016). In short, age-linked differences in multiple DTI-derived indicators of white matter suggest that the white matter tracts of older adults may be meaningfully different than the tracts of younger adults and, further, that these differences may contribute to age-related behavioral deficits.

Individual differences in RD and FA, especially in the left hemisphere, have also been linked to reading ability in children (Yeatman et al. 2012), language production in younger adults (For review see Dick et al. 2014), and even vocabulary knowledge in older adults (Teubner-Rhodes et al. 2016). However, only a few studies have examined the contribution of these factors to age-related differences in language production. Stamatakis and colleagues (2011) found that FA along dorsal, frontal tracts, such as the bilateral superior longitudinal fasciculus (SLF), as well as more ventral tracts such as the bilateral inferior longitudinal fasciculus (ILF), were positively correlated with accuracy in naming famous individuals. Moreover, they found a negative correlation between FA and age-related word finding failures, specifically along the bilateral posterior SLF (2011), which traverses regions associated with phonological processing (Hickok and Poeppel 2007). Stamatakis and colleagues also observed a significant leftward asymmetry, with more age-related differences in the left hemisphere. Together, these results suggest that white matter in the left dorsal language network (Dick et al. 2014), specifically the SLF, contribute to age-related differences in naming ability. Similarly, work by Madhavan and colleagues linked FA along the left SLF to several clinical measures of naming (e.g., Boston Naming Test; Madhavan et al. 2014). In line with theoretical models of language production (Dick et al. 2014; Hickok and Poeppel 2007), evidence suggests that some tracts in the ventral language network, specifically the left uncinate, (De Zubicaray et al. 2011) and left ILF (Kantarci et al. 2011; Stamatakis et al. 2011) also contribute to naming ability in older adults.

In addition to the empirically established relationships between naming ability and the SLF and ILF, theoretical models of language production also posit roles for two other fiber tracts—the frontal aslant tract (FAT) and the middle longitudinal fasciculus (MDLF). The FAT, which is a dorsal-frontal tract, is thought to be involved in the executive functions and working memory processes (Catani et al. 2012) necessary for language production (Rizio and Diaz 2016; Catani et al. 2013). The MDLF, a more ventral tract, is thought to be involved in mapping meaning (Saur et al. 2008), but the MDLF’s involvement in language remains somewhat controversial and needs to be more thoroughly evaluated (Dick et al. 2014). Moreover, the language literature to date has focused extensively on interpreting a single DTI metric at a time (i.e., only RD or only FA). Yet, both metrics have value. RD is particularly informative in older adult samples as an index of transverse diffusion (Davis et al. 2009; Bennett et al. 2010) while FA provides a more complete description of diffusion patterns and underlying structure. Thus, this study aims to examine the structural underpinnings of age-associated language deficits by linking naming to a more thorough characterization of age-associated differences in diffusion by using both RD and FA to index potential white matter differences in myelination and axon ultrastructure.

Specifically, we examined the neural underpinnings of age-associated language deficits in the context of the Transmission Deficit Hypothesis. The Transmission Deficit Hypothesis suggests that language difficulties stem from signal transmission failures in the phonological system. While transmission failures occur throughout the brain, failures in phonological connections are more likely to result in a behavioral deficit, such as difficulty naming objects, due to the one-to-one mapping between meaning and sound (i.e., the ordered sounds which make up a given word are only associated with that particular word). Semantic deficits, however, occur less often because that system is more redundant and interconnected (i.e., if one signal transmission fails in the semantic system, the signal may be conveyed via other connections; Burke et al. 1991). Indeed, this hypothesis is borne out in behavioral studies of language in which older adults show deficits in phonological but not semantic aspects of language (Diaz et al. 2014b; Burke et al. 2004; Abrams et al. 2007; James and Burke 2000). Some studies have even shown age-related enhancements in semantic processing (e.g., semantic distractors cause more interference in older adults; Taylor and Burke 2002); and semantic priming is sometimes found to speed lexical decision and word pronunciation more with age (Laver and Burke 1993). Likewise, individuals at all ages are more likely to make errors with proper names and nouns (e.g., Penn State, Sara) which are instantiated in memory as a single, low-strength node, than they are to make errors in naming common nouns, such as occupations (James 2004). Moreover, such errors with proper noun naming increase with age more than errors associated with common nouns (James 2006).

While the Transmission Deficit Hypothesis is well supported in the behavioral literature, the neural substrates of age-related phonological deficits are not fully understood. Therefore, this study represents an important examination of how age-related differences in white matter tracts associated with phonological (i.e., the frontal aslant tract and superior longitudinal fasciculus) and semantic (i.e., middle longitudinal fasciculus and inferior longitudinal fasciculus) processes relate to language production. We used DTI to assess white matter integrity in older and younger adults and a Picture-Word Interference (PWI) task to assess language production ability. Specifically, participants were asked to name pictures while ignoring either phonologically- or semantically- related distractors to assess the impact of phonological and semantic information on language production separately. Consistent with previous research, we hypothesized that older and younger adults would have different white matter networks as indicated by higher RD and lower FA in older compared to younger adults. We also expected that older adults relative to younger adults would show poorer performance on this language production task. Lastly, if RD and FA decline more for phonological processes as predicted by the Transmission Deficit Hypothesis, RD and FA should be related to behavioral performance on the phonological but not the semantic task. That is, we expected to observe a brain-behavior relationship in tracts associated with phonological but not semantic aspects of language production behavior.

## Methods

### Participants

18 younger (18–32, mean age = 23.7, SD = 6.23, 10 females) and 19 older (60–79, mean age = 67, SD = 4.51, 15 females) adults participated in this experiment. All participants were community-dwelling, right-handed, monolingual English speakers. To minimize perceptual acuity differences between older and younger adults, all participants had normal or corrected-to-normal vision as measured by the Freiburg Visual Acuity and Contrast Test (Bach 1996). No participants reported a history of neurological, psychological, or major health disorders (e.g., diabetes, heart disease; Christensen et al. 1992). All participants scored at least 27 on the Mini Mental State Examination (MMSE; Folstein et al. 1975) to limit the likelihood of including individuals with mild cognitive impairment or dementia. All participants completed neuropsychological testing to assess basic cognitive skills such as speed, memory, executive function, and language. Across groups, participants did not differ in years of education, handedness or measures of anxiety or depression. Nor did they differ on forward or backward digit span, verbal fluency, immediate verbal recall, digit symbol accuracy, Stroop effect scores, or vocabulary. Demographic characteristics are presented in Table 1. All participants provided written informed consent and were paid for their participation. All experimental procedures were approved by the Institutional Review Board at the Pennsylvania State University.

**Table 1:** Demographic and neuropsychological characteristics of participants

	Mean (SD)	
	YA	OA
N	18	19
Age (Years)**	23.67 (4.51)	67.00 (6.23)
Phonological Reaction Time (ms)	1297.94 (255.47)	1352.65 (259.93)
Semantic Reaction Time (ms)	1403.90 (318.28)	1432.04 (258.97)
Phonological Accuracy*	76.66% (7.45%)	69.26% (10.54%)
Semantic Accuracy*	72.00% (8.83%)	65.00% (8.76%)
Education (Years)	16.56 (2.85)	16.68 (2.21)
MMSE (out of 30)*	29.33 (0.91)	28.63 (1.07)
Forward Digit Span (out of 9)	7.22 (1.11)	7.16 (1.34)
Backward Digit Span (out of 8)	5.39 (1.33)	4.74 (1.19)
Verbal Fluency (Total)	66.11 (16.37)	68.72 (15.11)
Nonverbal Working Memory (RT; ms)**	839.49 (145.19)	1121.24 (363.23)
Immediate Recall (out of 16)	12.00 (1.85)	11.32 (1.70)
Delayed Recall (out of 16)**	11.44 (2.25)	9.11 (2.51)
Simple Speed of Processing (RT; ms)**	262.20 (33.48)	284.07 (43.83)
Complex Speed of Processing (RT; ms)	278.17 (21.96)	342.71 (79.28)
Digit Symbol (RT; ms)	1254.86 (245.94)	1831.72 (337.76)
Stroop Effect (ms)	9.1 (30.49)	94.03 (67.50)
Vocabulary (out of 66)	55.00 (6.25)	56.53 (5.50)
Author Recognition (out of 76)**	15.72 (8.51)	34.79 (15.05)
Magazine Recognition (out of 78)**	13.33 (6.69)	23.42 (7.46)

Significant difference between younger and older adults at *p < .05 and ** *p* < .005

While the absolute difference between younger and older adults’ Stroop Effect scores is large, a t-test did not reveal significant group differences due to the high variability in scores within both age groups

### Procedure

Both structural and functional Magnetic Resonance Imaging (MRI) data were collected. During the functional data collection, participants completed a PWI task in which they overtly named pictures that were accompanied by semantic, phonological, unrelated, or neutral distractors. This allowed us to specifically examine the role of phonological or semantic information in language production. The PWI task has been used extensively to study language ability in both younger and older adults (Alario et al. 2000; Rizio et al. 2017; Diaz et al. 2014a; Glaser and Düngelhoff 1984). See Rizio, Moyer, and Diaz for additional details and fMRI results (2017). Prior to scanning, participants practiced overt picture naming in a scanner simulator to familiarize them with the MR environment and to practice minimizing head movement while speaking. Importantly, participants were not familiarized with target words or pictures before entering the MRI. During the MRI session, overt verbal responses were recorded and filtered using an MR-compatible fiber optic microphone system (Optoacustics Ltd.,Or-Yehuda, Israel).

### Stimulus materials

**Fig. 1:**
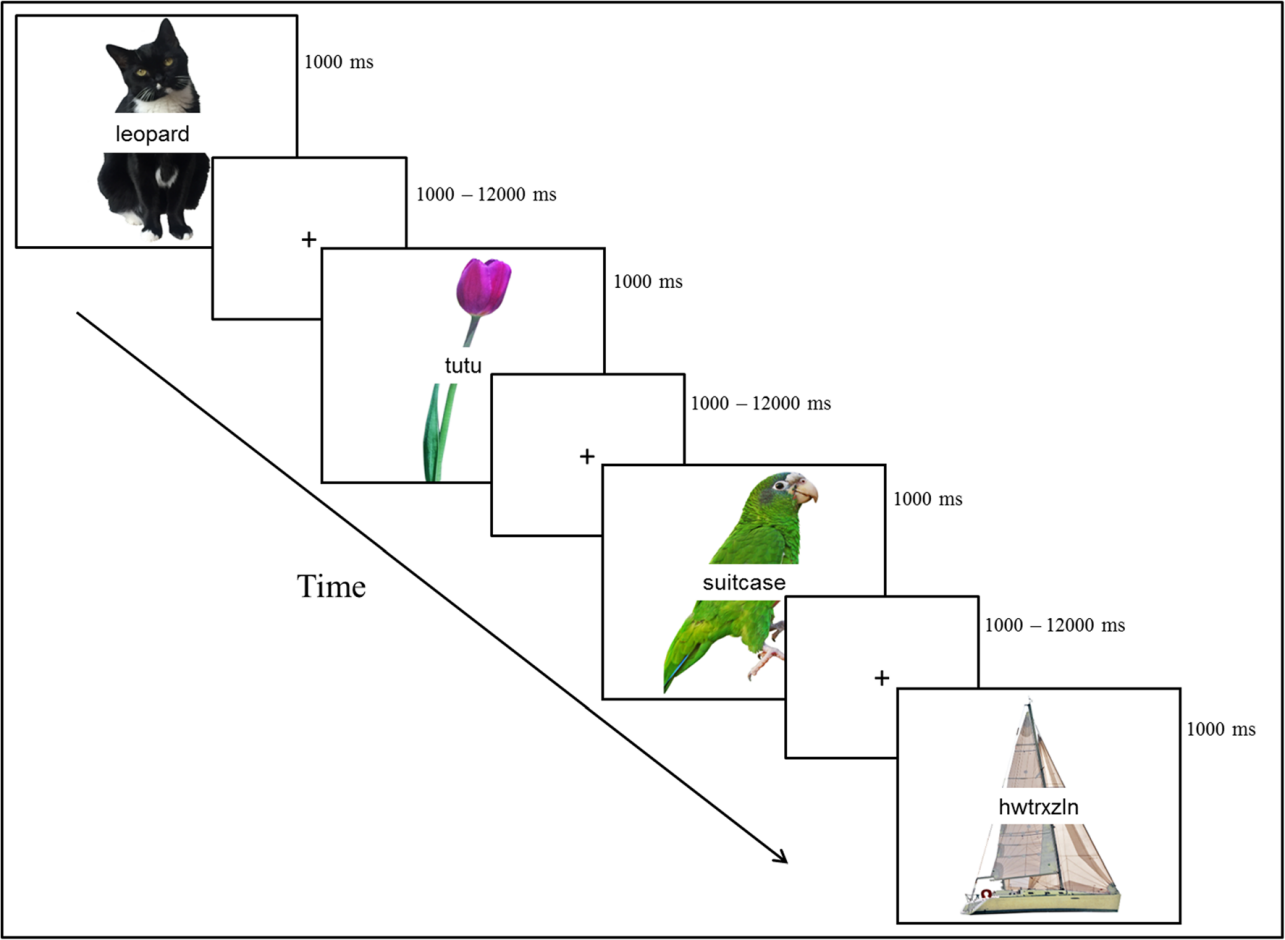
In the Picture-Word Interference (PWI) Task, participants were instructed to name the picture and ignore the superimposed word which was either semantically or phonologically related to the word or an unrelated word or letter string. This study will examine only the semantic and phonological conditions. Figure partially reproduced with permission from (Rizio et al. 2017)

Stimuli for the PWI paradigm included 240 colored images of common, concrete nouns such as animals, clothing, food, and household items from two normed picture databases (Brodeur et al. 2014; Moreno-Martinez and Montoro 2012) with a superimposed written distractor word (See Fig. 1).

For the present investigation, we focus on the semantic and phonological conditions. Lexical characteristics of the stimuli were matched across condition and details can be found in Rizio et al. (2017). Participants were instructed to name the target picture, but ignore the distractor word, and to respond as quickly as possible while still responding accurately. A fixation cross was presented between each stimulus presentation [inter-stimulus interval (ISI) range = 1–12 s, average ISI = 4 s]. ISIs were optimized with Optseq2 (Dale 1999). Trials were pseudorandomized so that no more than three items from the same distractor condition appeared in a row. Each of four runs (315 s) began and ended with the presentation of a fixation cross.

### Acquisition of MRI data

**Fig. 2:**
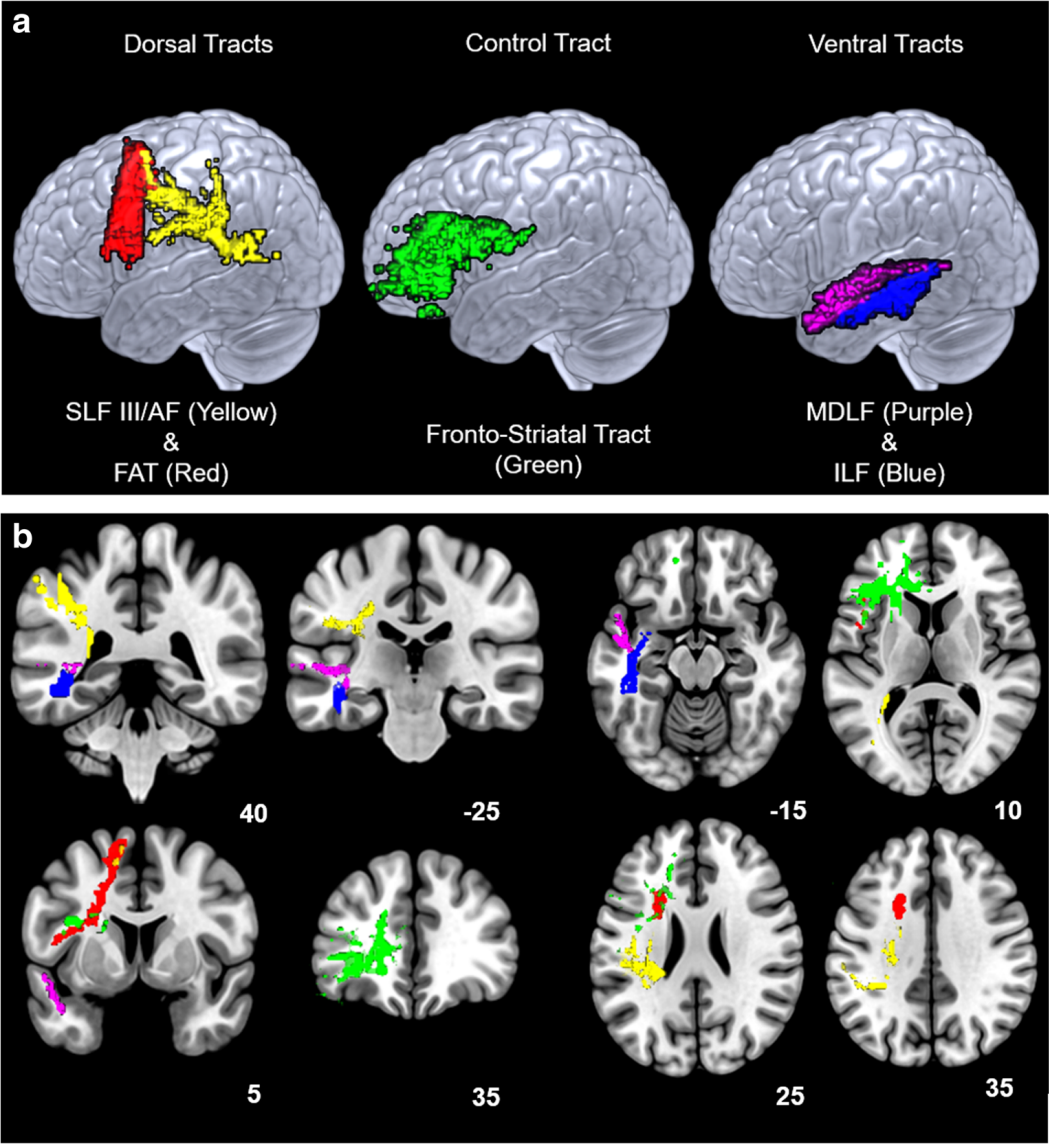
Representative examples of successful streamlines of white matter tracts from one participant. **a**. A 3D rendering of representative tracts on a lateral view. **b**. Coronal and axial slice views of the same tracts. Similar tract models were created separately for each participant in this study

Data were acquired using a 3 T Siemens Prisma Fit MRI scanner with a 20-channel head coil. Diffusion weighted data were acquired from 36 directions (62 interleaved contiguous slices, voxel size = 2 mm^3^, FOV = 240 mm^2^, TR = 10,000 ms; TE = 89 ms, echo spacing = .78 ms, flip angle = 90°, PE acceleration factor = 2, PE = AP, fat saturation was used). Diffusion weighted data were visually inspected for motion and artifacts using DTIprep (Oguz et al. 2014). The diffusion tensor model was fit to the data using DTIfit and crossing fibers were modeled using Bedpostx (Behrens et al. 2003; Behrens et al. 2007). Five separate tracts were then modeled using Probtrackx2 (Behrens et al. 2003; Behrens et al. 2007) in FSL (Smith et al. 2004). In each participant, tracts were modeled by sending out 5000 streamlines from each neuroanatomically defined seed region to a corresponding neuroanatomically defined target region (see online resource 1). To ensure fidelity of the data, tracts were also modeled in the reverse direction. That is, streamlines were sent from seed regions to target regions and from target regions to seed regions. Only those streamlines obtained in both tracking directions were retained for further analyses. The modeled tracts included two tracts of the dorsal language network, the superior longitudinal fasciculus-III (SLF)/ arcuate fasciculus (AF), the frontal aslant tract (FAT); two tracts of the ventral language network, the middle longitudinal fasciculus (MDLF), the inferior longitudinal fasciculus (ILF); and a comparison tract not associated with the dorsal or ventral language network, the fronto-striatal tract (see Fig. 2). Each tract was modeled separately in the left hemisphere of each participant given that the leftward lateralization of language remains stable across life (Stamatakis et al. 2011). These models were then used as masks to extract mean RD and mean FA along each tract.

Because there are theoretical relationships (Dick et al. 2014), as well as statistical collinearity among tracts, composite scores were created to reduce the number of comparisons being made. Specifically, SLF/AF and FAT tract values were averaged together to create a composite measure of dorsal tract integrity. Likewise, MDLF and ILF tract integrity values were averaged together to create a composite measure of ventral tract integrity.

### Data analysis

T-tests were used to test for group differences between older and younger adults on measures of RD, FA, and Reaction Time and Accuracy on the PWI task. The association between RD and performance on the PWI task (i.e., reaction time and accuracy) was assessed with bivariate correlations. We tested these brain-behavior relationships between FA and RD along dorsal tracts, ventral tracts, and the fronto-striatal tract. One-tailed Fisher R to Z transformations were then used to assess differences in the strength of correlations across tracts. Multivariate regression was used to test the effect of RD and FA, respectively, on PWI task performance with age as a covariate in each model. Separate regression models were built for dorsal, ventral, and fronto-striatal tracts.

## Results

There were no significant group differences between older and younger adults’ reaction times but older adults had significantly lower phonological accuracy (*t*(35) = 2.45, *p* = .02) and semantic accuracy (*t*(35) = 2.42, *p* = .02), compared to younger adults. Means and standard deviations of DTI metrics are reported in Table 2. In line with previous literature, older adults had significantly higher RD along dorsal (*t*(35) = 3.96, *p* < .001), ventral (*t*(35) = 3.03, *p* = .005) and fronto-striatal (*t*(35) = 4.56, *p* < .001) tracts, and lower FA along dorsal (*t*(35) = 6.37, *p* < .001), ventral (*t*(35) = 4.00, *p* < .001) and fronto-striatal (*t*(35) = 5.77, *p* < .001) tracts.

**Table 2:** Average RD and FA in younger and older adults

	Mean (SD)	
	YA	OA
N	18	19
Dorsal RD**	5.50 × 10^−4^ (.16 × 10^−4^)	5.90 × 10^−4^ (.39 × 10^−4^)
Ventral RD**	5.77 × 10^−4^ (.17 × 10^−4^)	6.01 × 10^−4^ (.30 × 10^−4^)
Fronto-Striatal RD**	5.51 × 10^−4^ (.14 × 10^−4^)	5.91 × 10^−4^ (.35 × 10^−4^)
Dorsal FA**	4.53 × 10^−1^ (.12 × 10^−1^)	4.22 × 10^−1^ (.17 × 10^−1^)
Ventral FA**	4.36 × 10^−1^ (.09 × 10^−1^)	4.17 × 10^−1^ (.18 × 10^−1^)
Fronto-Striatal FA**	4.30 × 10^−1^ (.12 × 10^−1^)	4.01 × 10^−1^ (.17 × 10^−1^)

Significant difference between younger and older adults, ** *p* < .005

Collapsing across age groups, there was a significant main effect of RD on phonological accuracy when RD was measured along dorsal (*r* = −.35, *p* < .05), ventral (*r* = −.34, *p* < .05), and fronto-striatal (*r* = −.33, *p* < .05) tracts, such that higher RD predicted lower phonological accuracy. Post-hoc Fisher R to Z tests comparing the strength of the relationship between phonological accuracy and RD along each tract indicated that the RD-accuracy relationship was comparable across tracts (*p* > .05). FA along dorsal tracts was positively correlated with phonological accuracy (*r* = .43, *p* < .01) but FA along ventral (*r* = .22, *p* > .05) and fronto-striatal (*r* = .16, *p* > .05) tracts was not correlated with phonological accuracy. Again, post-hoc Fisher R to Z tests comparing the strength of relationship between phonological accuracy and FA along each tract indicated that the FA-accuracy relationship was comparable across tracts (*p* > .05). Semantic accuracy was not significantly related to RD or FA along dorsal, ventral, or fronto-striatal tracts (*p* > .05).

When covarying for the effect of age group in multivariate regression models, each of these main effects (the effect of dorsal, ventral, and fronto-striatal RD and the effect of dorsal FA on phonological accuracy) was attenuated to non-significant levels (see Table 3). To explore the possibility of a moderation effect, we reanalyzed each model with an additional age by white matter interaction term. However, none of the interaction terms were significant. We also ran a post-hoc analysis of achieved power which revealed that this study was underpowered to detect small effect sizes but was appropriately powered to detect moderate to large effects.

**Table 3:** Multivariate regression models predicting phonological accuracy

	B (unstandardized)	SE B	*β*	*T*	*p*
Dorsal tracts-RD					
RD	−540.98	508.06	−0.20	−1.07	0.29
Age group	−0.05	0.04	−0.27	−1.45	0.16
Ventral tracts-RD					
RD	−718.46	621.00	−0.20	−1.16	0.26
Age group	−0.06	0.03	−0.29	−1.67	0.10
Fronto-striatal tracts-RD					
RD	−475.72	580.97	−0.16	−0.82	0.42
Age group	−0.06	0.04	−0.29	−1.45	0.16
Dorsal tracts-FA					
FA	1.44	1.03	.32	1.39	.17
Age group	−.03	.04	−.15	−.67	.51

Table 3 displays results of our multivariate regression analysis. B (unstandardized) and β (standardized) provide an indication of absolute and relative effect size of white matter on phonological accuracy. SE B provides an indication of the variability in each of the predictors while *T* characterizes this effect size for each predictor in terms of a standard *T* distribution. Lastly, *p* indicates the probability of finding an effect as large or larger than the one observed in this sample by chance. In this case, we set our alpha level at *p* = .05, to indicate 95% confidence that the observed effect was not due to chance. None of the effects in the multivariate models surpassed this threshold

## Discussion

Despite widely reported age-related language production difficulties, semantic aspects of language are relatively preserved. As such, this study sought to compare the structural underpinnings of language production in older and younger adults. We used a Picture-Word Interference (PWI) task in which participants overtly produced the names of pictures while ignoring phonologically- or semantically-related distractor words, to measure the effects of phonological and semantic information on language production behavior. We also incorporated multiple metrics derived from Diffusion Tensor Imaging (DTI) to measure participants white matter myelination and ultrastructure. In line with previous literature on language production (Burke and Shafto 2004), our study found older adults had lower accuracy than younger adults on the PWI task of language production. Also, as predicted, we found that older adults had higher RD and lower FA along dorsal, ventral, and fronto-striatal tracts, confirming previous reports of age-related differences in diffusion patterns between older and younger adults in this sample. As such, this study provides two conceptual replications. First, we replicated the finding that older adults have more language production deficits than younger adults (Burke and Shafto 2004). Second, we replicated the finding that age is related to differences in diffusion patterns indicative of white matter across language-associated brain regions (Madhavan et al. 2014; Stamatakis et al. 2011)

In addition to conceptual replications, this study also provides a novel contribution to our understanding of the structural underpinnings of language production; this is the first study to compare both RD and FA across dorsal and ventral language tracts and a control tract in younger and older adults. In so doing, we found that lower RD — which we interpret as an index of myelination — along dorsal, ventral, and fronto-striatal tracts predicted higher phonological accuracy across groups. Along dorsal tracts, individual differences in FA — which we interpret as an index of overall fiber coherence — also predicted performance on a phonological language production task. Post-hoc Fisher R to Z tests indicated that dorsal, ventral, and fronto-striatal tract RD and FA were all comparably related to phonological behavior, highlighting the importance of white matter myelination and ultrastructure across, rather than within, networks. These results suggest that individual differences in white matter exist across the brain and may influence language production performance. However, when considering multiple DTI metrics, only in dorsal tracts did we observe a change in both RD and FA. This implies that, while myelin differences across the white matter system are evident, individual differences in white matter fiber coherence along dorsal tracts in particular may be associated with the phonological (Stamatakis et al. 2011; Hickok and Poeppel 2007; Dick et al. 2014) and working memory (Rizio and Diaz 2016) processes necessary for language production.

Considering both RD and FA, the findings of this study are consistent with much of the previous literature on white matter and naming; Stamatakis and Madhavan have both linked naming behavior in older adults to FA along the posterior portion of the bilateral SLF (Stamatakis et al. 2011; Madhavan et al. 2014). Likewise, Rizio and Diaz (2016) linked FA along the bilateral FAT to working memory and have put forth that this may play a role in driving age-associated language deficits. Notably, neither RD nor FA along ventral tracts was linked to semantic aspects of language production as would be expected based on the work of De Zubicaray and Kantarci, (De Zubicaray et al. 2011; Kantarci et al. 2011). This may be because our operationalization of the ventral stream tracts was grounded more in anatomy than function (Dick et al. 2014; Saur et al. 2008). We suggest that future research may find different results when examining alternate ventral language network tracts (i.e., uncinate). However, it is also possible that there were no observed links between ventral white matter and language production because of power constraints. In fact, observed power was only above 80% for medium and large effects (e.g., the effect of dorsal FA on phonological RT). Despite power concerns, these findings still provide evidence that age-related myelin deficits across the brain play a role in driving age-related cognitive deficits (Bennett and Madden 2014) and that age-related language deficits may be particularly linked to differences in ultrastructural coherence along dorsal tracts (Stamatakis et al. 2011).

An additional reason we may have found differing results from some previous studies is our novel approach of utilizing both RD and FA. Indeed, different conclusions would be drawn if the RD and FA metrics were interpreted in isolation. For instance, when structure was measured by RD alone, relationships between structure and behavior (phonological accuracy) were evident across all tracts (dorsal, ventral, and fronto-striatal tracts), suggesting a global and non-specific relationship between our measure of white matter myelination and language ability. If only the FA results were interpreted, the conclusions of this study would be that only overall structure coherence along dorsal tracts contributes to phonological behavior. Importantly, we chose to interpret our RD and FA results in combination because the interpretation of multiple DTI metrics is considered a more robust method (Jones et al. 2013). Moreover, interpreting both RD and FA provides additional information about what diffusion differences are of consequence. For instance, we found that both RD and FA along dorsal tracts were related to behavior indicating that, along dorsal tracts, both myelin and ultrastructural coherence of these tracts are related to phonological processes. Conversely, along the ventral and fronto-striatal tracts, we found relationships between behavior and RD but not FA. This suggests that changes in diffusion along the radius of the tensor (i.e., myelination) may be related to phonological processes but the ultrastructural fiber coherence along these ventral and fronto-striatal tracts may not play a role in language production. We do caution, however, that while FA is likely to reflect difference in fiber coherence, there may be other factors influencing this metric which should be evaluated further in future research. The recent development of orientation dispersion index, a metric for resolving fiber coherence, specifically, may aid future research in disentangling the more specific contribution of neurite density and fiber orientation to age-related white matter decline. In sum, our results indicate that global white matter differences may play some role in language ability but that dorsal tracts are particularly closely related to phonological behavior as evidenced by the importance of both myelination and ultrastructural fiber organization along these tracts. Future work should specifically investigate how the SLF-III/AF and the FAT relate to language production but the distal role of ventral tracts should not be discounted.

One limitation of this study concerns the interpretation of our behavioral findings. According to the Transmission Deficit Hypothesis, when phonological cues are provided, as in the phonological condition of this manipulation, a facilitation effect should be observed. That is, higher accuracy and shorter reaction times. Conversely, in the semantic condition, the additional information should create interference and induce poorer performance as measured by lower accuracy and longer reaction times (Burke et al. 1991). Yet, we found that overall, both conditions had similar accuracies, and that older adults had lower accuracies compared to younger adults on both the phonological and semantic conditions of the PWI task. However, we do not believe the task was too easy as neither older nor younger adults performed at ceiling. Another possibility is that our results may have been influenced by older and younger adults’ approaching the task in systematically different ways because of cohort effects. For instance, older adults have more extensive vocabulary knowledge than younger adults (Verhaeghen 2003) and have been shown to produce more lexically diverse speech (Kavé et al. 2009). In other words, older adults may have selected more cognitively demanding words or may have been selecting from a larger lexicon compared with younger adults. However, we did not observe statistically different vocabulary scores for our groups and the percentage of acceptable alternatives that were produced was the same across groups. Therefore, though there is some uncertainty about differences in strategy use, our data suggest similarity in the two groups’ overall approach to the task. Yet another possibility is that the lack of difference between the semantic and phonological conditions is due to the relatively poor performance of our younger adult sample. That is, despite differences in accuracy, both older and younger adults exhibited comparable reaction times. In direct tests of speed, older adults had significantly longer simple reaction times compared to younger adults. However, both groups had comparable complex reaction times. Comparisons with prior work from our group and others, suggests that the lack of differences was due to relatively poor performance on the part of younger adults rather than well-preserved performance in older adults (Rizio et al. 2017).

Therefore, in the context of these limitations, we take our results as partial support for the Transmission Deficit Hypothesis (Burke and MacKay 1997). Specifically, we find evidence suggesting significant brain-behavior relationships exist between dorsal white matter tracts and phonological aspects of language production. We do not find evidence of a relationship between ventral white matter tracts and semantic aspects of language production. This pattern of results aligns with the Transmission Deficit Hypothesis assertion that brain-behavior relationships should be evident in the phonological but not semantic domain. However, we qualify our interpretation as only partially supporting the Transmission Deficit Hypothesis because, in post-hoc tests, we did not find a significant difference in the effect sizes of the brain-behavior relationships between the two conditions. In other words, we find evidence for the existence of phonological brain-behavior relationships and not semantic brain-behavior relationships but cannot provide evidence for the Transmission Deficit Hypothesis assertion that phonological brain-behavior relationships are larger in magnitude than semantic brain-behavior relationships. Though this partial support for the Transmission Deficit Hypothesis concerns effects observed when collapsing across groups, it is also important to note the role of age. When age was entered into regression models, the effects of RD and FA were attenuated to insignificant levels. This indicates that individual variation in white matter significantly overlaps with the individual variation in language ability explained by age. Ultimately, this effect adds further support to the idea that white matter changes with age and these changes have important implications for language production ability.

In summary, this study provides convergent evidence that age-related differences in RD and FA, which are typically interpreted to be indicative of white matter, are linked to age-related language production deficits. Moreover, this is the first study, to the authors’ knowledge, to use both RD and FA along dorsal and ventral tracts to test language production in older adults. In so doing, this study links age-related white matter disconnection to behavioral explanations of preserved comprehension ability and diminished production ability in later life, provides a replication of previous DTI studies on language and aging, and gives novel insight into the relationship between age-related language production deficits and white matter disconnection.

## Electronic supplementary material

ESM 1 (PDF 183 kb)

## References

[CR1] Abrams L, Trunk DL, Merrill LA (2007). Why a superman cannot help a tsunami: activation of grammatical class influences resolution of young and older adults' tip-of-the-tongue states. Psychology and Aging.

[CR2] Alario XF, Segui J, Ferrand L (2000). Semantic and associative priming in picture naming. The Quarterly Journal of Experimental Psychology Section A.

[CR3] Bach M (1996). The Freiburg visual acuity test--automatic measurement of visual acuity. Optometry and Vision Science.

[CR4] Basser PJ (1995). Inferring microstructural features and the physiological state of tissues from diffusion-weighted images. NMR in Biomedicine.

[CR5] Basser PJ, Pierpaoli C (1996). Microstructural and physiological features of tissues elucidated by quantitative-diffusion-tensor MRI. Journal of Magnetic Resonance. Series B.

[CR6] Behrens TE, Woolrich MW, Jenkinson M, Johansen-Berg H, Nunes RG, Clare S (2003). Characterization and propagation of uncertainty in diffusion-weighted MR imaging. Magnetic Resonance in Medicine.

[CR7] Behrens TE, Berg HJ, Jbabdi S, Rushworth MF, Woolrich MW (2007). Probabilistic diffusion tractography with multiple fibre orientations: What can we gain?. Neuroimage.

[CR8] Bennett IJ, Madden DJ (2014). Disconnected aging: Cerebral white matter integrity and age-related differences in cognition. Neuroscience.

[CR9] Bennett IJ, Madden DJ, Vaidya CJ, Howard DV, Howard JH (2010). Age-related differences in multiple measures of white matter integrity: a diffusion tensor imaging study of healthy aging. Human Brain Mapping.

[CR10] Brodeur MB, Guerard K, Bouras M (2014). Bank of Standardized Stimuli (BOSS) phase II: 930 new normative photos. PLoS One.

[CR11] Burke DM, MacKay DG (1997). Memory, language, and ageing. Philosophical Transactions of the Royal Society: Biological Sciences.

[CR12] Burke DM, Shafto MA (2004). Aging and language production. Current Directions in Psychological Science.

[CR13] Burke DM, Shafto MA (2008). Language and aging. The handbook of aging and cognition.

[CR14] Burke DM, MacKay DG, Worthley JS, Wade E (1991). On the tip of the tongue: what causes word finding failures in young and older adults?. Journal of Memory and Language.

[CR15] Burke DM, Locantore JK, Austin AA, Chae B (2004). Cherry pit primes Brad Pitt: Homophone priming effects on young and older adults' production of proper names. Psychological Science.

[CR16] Catani M, Dell’Acqua F, Vergani F, Malik F, Hodge H, Roy P (2012). Short frontal lobe connections of the human brain. Cortex.

[CR17] Catani M, Mesulam MM, Jakobsen E, Malik F, Martersteck A, Wieneke C, Thompson CK, Thiebaut de Schotten M, Dell’Acqua F, Weintraub S, Rogalski E (2013). A novel frontal pathway underlies verbal fluency in primary progressive aphasia. Brain.

[CR18] Christensen KJ, Moye J, Armson RR, Kern TM (1992). Health screening and random recruitment for cognitive aging research. Psychology and Aging.

[CR19] Dale AM (1999). Optimal experimental design for event-related fMRI. Human Brain Mapping.

[CR20] Davis SW, Dennis NA, Buchler NG, White LE, Madden DJ, Cabeza R (2009). Assessing the effects of age on long white matter tracts using diffusion tensor tractography. Neuroimage.

[CR21] De Zubicaray GI, Rose SE, McMahon KL (2011). The structure and connectivity of semantic memory in the healthy older adult brain. Neuroimage.

[CR22] Diaz MT, Hogstrom LJ, Zhuang J, Voyvodic JT, Johnson MA, Camblin CC (2014). Written distractor words influence brain activity during overt picture naming. Frontiers in Human Neuroscience.

[CR23] Diaz MT, Johnson MA, Burke DM, Madden DJ (2014). Age-related differences in the neural bases of phonological and semantic processes. Journal of Cognitive Neuroscience.

[CR24] Dick AS, Bernal B, Tremblay P (2014). The language connectome: new pathways, new concepts. Neuroscientist.

[CR25] Feyereisen P (1997). A meta-analytic procedure shows an age-related decline in picture naming: comments on Goulet, Ska, and Kahn (1994). Journal of Speech, Language, and Hearing Research.

[CR26] Fjell AM, Sneve MH, Grydeland H, Storsve AB, Amlien IK, Yendiki A, Walhovd KB (2017). Relationship between structural and functional connectivity change across the adult lifespan: A longitudinal investigation. Human Brain Mapping.

[CR27] Folstein MF, Folstein SE, McHugh PR (1975). "Mini-mental state". A practical method for grading the cognitive state of patients for the clinician. Journal of Psychiatric Research.

[CR28] Gazes Y, Bowman FD, Razlighi QR, O'Shea D, Stern Y, Habeck C (2016). White matter tract covariance patterns predict age-declining cognitive abilities. Neuroimage.

[CR29] Glaser WR, Düngelhoff F-J (1984). The time course of picture-word interference. Journal of Experimental Psychology: Human Perception and Performance.

[CR30] Hedden T, Schultz AP, Rieckmann A, Mormino EC, Johnson KA, Sperling RA, Buckner RL (2016). Multiple brain markers are linked to age-related variation in cognition. Cerebral Cortex.

[CR31] Hickok G, Poeppel D (2007). The cortical organization of speech processing. Nature Reviews Neuroscience.

[CR32] James LE (2004). Meeting Mr. farmer versus meeting a farmer: specific effects of aging on learning proper names. Psychology and Aging.

[CR33] James LE (2006). Specific effects of aging on proper name retrieval: now you see them, now you Don't. The Journals of Gerontology Series B: Psychological Sciences and Social Sciences.

[CR34] James LE, Burke DM (2000). Phonological priming effects on word retrieval and tip-of-the-tongue experiences in young and older adults. Journal of Experimental Psychology: Learning, Memory, and Cognition.

[CR35] Jones DK, Knösche TR, Turner R (2013). White matter integrity, fiber count, and other fallacies: the do's and don'ts of diffusion MRI. Neuroimage.

[CR36] Kantarci K, Senjem M, Avula R, Zhang B, Samikoglu A, Weigand S (2011). Diffusion tensor imaging and cognitive function in older adults with no dementia. Neurology.

[CR37] Kavé G, Samuel-Enoch K, Adiv S (2009). The association between age and the frequency of nouns selected for production. Psychology and Aging.

[CR38] Kemper S, Kynette D, Norman S (1992). Age differences in spoken language. Everyday memory and aging.

[CR39] Laver GD, Burke DM (1993). Why do semantic priming effects increase in old age? A meta-analysis. Psychology and Aging.

[CR40] Madden DJ, Bennett IJ, Song AW (2009). Cerebral white matter integrity and cognitive aging: contributions from diffusion tensor imaging. Neuropsychology Review.

[CR41] Madhavan KM, McQueeny T, Howe SR, Shear P, Szaflarski J (2014). Superior longitudinal fasciculus and language functioning in healthy aging. Brain Research.

[CR42] Moreno-Martinez FJ, Montoro PR (2012). An ecological alternative to Snodgrass & Vanderwart: 360 high quality colour images with norms for seven psycholinguistic variables. PLoS ONE.

[CR43] Oguz, I., Farzinfar, M., Matsui, J., Budin, F., Liu, Z., Gerig, G., et al. (2014). DTIPrep: quality control of diffusion-weighted images. *Frontiers in Neuroinformatics, 8*(4). 10.3389/fninf.2014.00004. 10.3389/fninf.2014.00004 PMC3906573 24523693

[CR44] Ossher L, Flegal KE, Lustig C (2013). Everyday memory errors in older adults. Aging, Neuropsychology, and Cognition.

[CR45] Pierpaoli C, Jezzard P, Basser PJ, Barnett A, Di Chiro G (1996). Diffusion tensor MR imaging of the human brain. Radiology.

[CR46] Rizio AA, Diaz MT (2016). Language, aging, and cognition: frontal aslant tract and superior longitudinal fasciculus contribute toward working memory performance in older adults. Neuroreport.

[CR47] Rizio AA, Moyer KJ, Diaz MT (2017). Neural evidence for phonologically based language production deficits in older adults: an fMRI investigation of age-related differences in picture-word interference. Brain and Behavior.

[CR48] Ryan EB, Hummert ML, Boich LH (1995). Communication predicaments of aging: patronizing behavior toward older adults. Journal of Language and Social Psychology.

[CR49] Saur D, Kreher BW, Schnell S, Kümmerer D, Kellmeyer P, Vry M-S (2008). Ventral and dorsal pathways for language. Proceedings of the National Academy of Sciences.

[CR50] Smith SM, Jenkinson M, Woolrich MW, Beckmann CF, Behrens TE, Johansen-Berg H (2004). Advances in functional and structural MR image analysis and implementation as FSL. Neuroimage.

[CR51] Song S-K, Sun S-W, Ramsbottom MJ, Chang C, Russell J, Cross AH (2002). Dysmyelination revealed through MRI as increased radial (but unchanged axial) diffusion of water. Neuroimage.

[CR52] Stamatakis EA, Shafto MA, Williams G, Tam P, Tyler LK (2011). White matter changes and word finding failures with increasing age. PLoS One.

[CR53] Takahashi M, Hackney DB, Zhang G, Wehrli SL, Wright AC, O'Brien WT, Uematsu H, Wehrli FW, Selzer ME (2002). Magnetic resonance microimaging of intraaxonal water diffusion in live excised lamprey spinal cord. Proceedings of the National Academy of Sciences.

[CR54] Taylor JK, Burke DM (2002). Asymmetric aging effects on semantic and phonological processes: naming in the picture-word interference task. Psychology and Aging.

[CR55] Teubner-Rhodes S, Vaden KI, Cute SL, Yeatman JD, Dougherty RF, Eckert MA (2016). Aging-resilient associations between the arcuate fasciculus and vocabulary knowledge: microstructure or morphology. Journal of Neuroscience.

[CR56] Verhaeghen P (2003). Aging and vocabulary score: a meta-analysis. Psychology and Aging.

[CR57] Yeatman JD, Dougherty RF, Ben-Shachar M, Wandell BA (2012). Development of white matter and reading skills. Proceedings of the National Academy of Sciences.

