# Supplementary material for: White matter disconnection is related to age-related phonological deficits
Source: Brain Imaging Behav. 2019 Apr 2;14(5):1555–65. doi: 10.1007/s11682-019-00086-8 (PMC7034773; doi:10.1007/s11682-019-00086-8)
Supplement: Supplementary file 1 — (PDF 183 kb) [file 11682_2019_86_MOESM1_ESM.pdf]

White Matter Disconnection is Related to Age-Related Phonological Deficits

Sara B.W.Troutman[1] and Michele T. Diaz[1]

[1]Pennsylvania State University, University Park, PA, USA, 16802

Journal: Brain Imaging and Behavior

Corresponding author:

Michele T. Diaz, PhD

Department of Psychology

Pennsylvania State University

365 Moore Building, University Park, PA, USA, 16802

Tel: +1 814 863 1726

Fax: + 1 814 863 7002

e-mail: mtd143@psu.edu

ORCID: 0000-0001-7263-1694

	Control Tract	Semantic Tracts		Phonological Tracts	
	**frontal striatal tract (FS)**	**inferior longitudinal fasciculus (ILF)**	**middle longitudinal fasciculus (MDLF)**	**frontal aslant tract (FAT)**	**superior longitudinal fasciculus (SLF) III /arcuate fasciculus (AF)**
**Seed**	dorsolateral prefrontal cortex as defined as a 8mm sphere centered at X=22 Y=87 Z=38	temporal pole as defined by the Harvard Oxford Cortical Atlas thresholded at 25%	angular gyrus as defined by the Harvard Oxford Cortical Atlas thresholded at 25%	supplementary motor cortex (SMA) and pre-SMA as defined by the Harvard Oxford Cortical Atlas thresholded at 25%	inferior frontal gyrus pars operculus, as defined by Harvard Oxford Cortical Atlas thresholded at 25%
**Target**	caudate defined by the Harvard Oxford Atlas thresholded at 25%	temporo-occipital portion of the inferior temporal gyrus defined by the Harvard Oxford Cortical Atlas thresholded at 25%	temporal pole defined by the Harvard Oxford Cortical Atlas thresholded at 25%	inferior frontal gyrus pars operculus and pars triangulars as defined by the Harvard Oxford Cortical Alas thresholded at 25%	superior temporal gyrus defined by Harvard Oxford Cortical Atlas thresholded at 25%
